# Intrauterine smoke exposure deregulates lung function, pulmonary transcriptomes, and in particular insulin-like growth factor (IGF)-1 in a sex-specific manner

**DOI:** 10.1038/s41598-018-25762-5

**Published:** 2018-05-15

**Authors:** Stefan Dehmel, Petra Nathan, Sabine Bartel, Natalia El-Merhie, Hagen Scherb, Katrin Milger, Gerrit John-Schuster, Ali Oender Yildirim, Machteld Hylkema, Martin Irmler, Johannes Beckers, Bianca Schaub, Oliver Eickelberg, Susanne Krauss-Etschmann

**Affiliations:** 1 Comprehensive Pneumology Center (CPC-M), Institute of Lung Biology and Disease, Helmholtz Zentrum Muenchen, Member of the German Research Center for Lung Research (DZL), Neuherberg, Germany; 2 Early Life Origins of Chronic Lung Disease, Research Center Borstel, Leibniz Lung Center, Member of the German Research Center for Lung Research (DZL), Borstel, Germany; 3 Institute of Computational Biology, Helmholtz Zentrum Muenchen - German Research Centre for Environmental Health, Neuherberg, Germany; 4 Department of Internal Medicine V, University of Munich (LMU), Munich, Germany; 5 Department of Pathology and Medical Biology, GRIAC Research Institute, University of Groningen, University Medical Center Groningen, Groningen, Netherlands; 6 Institute of Experimental Genetics, Helmholtz Zentrum Muenchen, Neuherberg, Germany; 7 Chair of Experimental Genetics, Technische Universität München, Freising, Germany; 8 German Center for Diabetes Research (DZD), Neuherberg, Germany; 9 Pediatric Allergology Department of Pediatrics, Christian-Albrechts-Universitaet zu Kiel, Dr. von Hauner Children´s Hospital University Hospital, LMU Munich, Munich, Germany; 10 Division of Pulmonary Sciences and Critical Care Medicine, University of Colorado Anschutz Medical Campus, Aurora, CO, USA; 11 Institute for Experimental Medicine, Christian-Albrechts-Universitaet zu Kiel, Kiel, Germany

## Abstract

Prenatal exposure to tobacco smoke is a significant risk-factor for airway disease development. Furthermore, the high prevalence of pregnant smoking women requires the establishment of strategies for offspring lung protection. Therefore, we here aimed to understand the molecular mechanism of how prenatal smoke exposure affects fetal lung development. We used a mouse model recapitulating clinical findings of prenatally exposed children, where pregnant mice were exposed to smoke until c-section or spontaneous delivery, and offspring weight development and lung function was monitored. Additionally, we investigated pulmonary transcriptome changes in fetal lungs (GD18.5) by mRNA/miRNA arrays, network analyses and qPCR. The results demonstrated that prenatally exposed mice showed intrauterine and postnatal growth retardation, and impaired lung function. 1340 genes and 133 miRNAs were found to be significantly dysregulated by *in utero* smoke exposure, and we identified Insulin-like growth factor 1 (Igf1) as a top hierarchical node in a network analysis. Moreover, *Igf1* mRNA was increased in female murine offspring and in prenatally exposed children. These findings suggest that prenatal smoking is associated with a dysregulation of several genes, including *Igf1* in a sex-specific manner. Thus, our results could represent a novel link between smoke exposure, abberant lung development and impaired lung function.

## Introduction

Smoking during pregnancy is a recognized risk factor for low birth weight^1,2^ and impaired lung function in offspring^3–5^. In addition, even a sole exposure to maternal nicotine, a component of tobacco smoke, affects the metabolism and the structuaral development of the offspring lung^6^. Furthermore, there is evidence for increased risk of preschool wheeze^7–9^, asthma^8,9^ and COPD^10–12^ in the offspring. Despite this knowledge and intensive anti-tobacco campaigns in several countries, the number of women smoking during pregnancy remains high worldwide. These facts prompted demands for research on consequences of prenatal smoke exposure including downstream biological effects^13^.

Besides adverse health consequences of maternal smoking, it has been proposed that grandmaternal as well as grandpaternal smoking during pregnancy is sufficient to affect weight gain of the grandchildren, even if their mother was not smoking during her pregnancy^14,15^. Moreover, it was shown that grandmaternal smoking affects asthma susceptibility^16–18^ in grandchildren. These observations were supported by studies in rats where maternal perinatal s.c. nicotine induced airway hyperreactivity until the 3rd generation^19^. If these transgenerational effects hold true, even highly successful smoking cessation programs would take decades to become fully effective.

Various growth factors play a role during the process of fetal lung development^20–26^. As previous studies revealed, one of these factors is insulin-like growth factor (IGF), while it was shown that mice depleted from either *Igf1* or its receptor *Igf1r* demonstrated a failure in lung development^27,28^. In addition to this, few studies showed sex-specific differences in the IGF-system of the offspring following maternal smoking^29,30^. Therefore, it is important to develop means to protect the developing fetal lung as well as to understand the mechanisms of how cigarette smoke affects fetal and early postnatal lung development.

Thus, in this study we aimed to investigate the underlying molecular mechanisms of *in utero* smoke exposure and the resulting predisposition for chronic lung diseases. Along this line, we identified dysregulations of 1340 genes and 133 microRNAs in the fetal lung, while one central hierarchical node was *Igf1*. In addition, an increase of *IGF1* was also observed in school children with prenatal and early-life cigarette smoke-exposure history.

## Materials and Methods

For a detailed description of RNA isolation and qPCR please refer to the Supplementary Information.

### Animal protocols

The study was conducted according to the guidelines for the use and care of laboratory animals according to the Federal Act on the Protection of Animals (Germany). The study was approved by the Government of the District of Upper Bavaria (GZ. 55.2-1-54-2532-91-11). Balb/c mice, obtained from Charles River (Sulzfeld, Germany), were housed in a specific pathogen-free facility with a 12 h day-night cycle at a constant temperature and humidity. The mice were provided with standard rodent chow and water *ad libitum*. Eight-week old virgin females were mated 1:1 with proven male breeder for 24 h (defined as embryonic day (ED) 0.5). To allow proper implantation of the fertilized oocyte, mice were exposed to smoke or filtered air (see below) only from gestational day (GD) 2.5 until spontaneous delivery or until GD 17.5 followed by caesarean section at GD 18.5. Since the successful fertilization wasn’t possible at the start of exposure, non-pregnant mice were equally exposed to cigarette smoke (CS) until pregnancy could be excluded through maternal weight control. For caesarean section (c-section), pregnant mice were anaesthetized with Ketamine/Xylazine on GD 18.5. Uteri were visually inspected for resorbed fetuses and the uterine position of each fetus was recorded. Fetuses were weighed and sacrificed by i.p. injection of pentobarbital for organ removal. Fetal lungs were carefully removed, weighed (Mettler Toledo, Greifensee, Switzerland), and stored in RNAlater (Ambion, Austin, USA) at 4 °C overnight and then at −80 °C until RNA isolation.

### Cigarette smoke exposure protocol

Mainstream CS served as a surrogate for active smoking and was generated as previously described^31^. Briefly, smoke was generated from 3R4F Research Cigarettes (Tobacco Research Institute, University of Kentucky, Lexington, KY) and drawn into the exposure chamber with a membrane pump. Mice were exposed to 10 cigarettes for 50 min every morning until c-section or spontaneous delivery. Total particulate matter (TPM) concentrations were monitored by drawing an air sample from the exposure chamber through a quartz fiber filter (at 3^rd^, 6^th^, and 9^th^ cigarette, each day during the exposure). The TPM mass concentration was obtained by gravimetric analysis of the filters before and after exposure and was then related to the chamber volume.

### Characterization of pregnant mice

After mating, maternal weight was monitored daily until delivery. Urine was collected four hours after the last smoke exposure and cotinine was quantified by ELISA (Calbiotech, Spring Valley, US). For an independent confirmation of smoke exposure, carbon monoxide hemoglobin (CO-Hb) was determined in venous blood obtained from the retrobulbar venous plexus in anesthetized animals 30 min after the last cigarette (Hemoxymeter (OSM3, Radiometer, Copenhagen).

### Characterization of murine offspring

Body weight was recorded daily in spontaneously delivered offspring until postnatal day (PND) 56. Lung function was performed at PND21. A total of 303 offspring were analyzed (Supplementary Table S1). Since body weight was confounded by litter size, only pups from litters above the median (>6) were included in the final molecular analyses. To further avoid ill-defined seasonal effects, animals obtained from different experimental batches at different seasons were included in the molecular analyses.

### Lung function analyses

Following intraperitoneal anesthesia (ketamine 140 mg/kg; xylazine 7 mg/kg), mice were tracheostomized, intubated (19 G tube) and placed on a warming plate. Pancuronium (1 mg/kg, i.p.) was added to avoid spontaneous breathing during examination. For baseline lung function measurements, mice were ventilated with a tidal volume of 11 ml/kg at a frequency of 150 breaths/minute with a positive end-expiratory pressure of 2 cm H_2_O on a computer controlled ventilator (FlexiVent®, SCIREQ©, Montreal, QC, Canada). The following perturbations were performed: Maximal Vital Capacity (MVC) maneuver, single compartment (snapshot), constant phase model (Primewave-8), and pressure-volume loops with constantly increasing pressure (PVr-P). All perturbations were performed one-by-one until three acceptable measurements (coefficient of determination > 0.95) were recorded in each individual subject, of which an average was calculated. Due to a body weight difference in male and female mice, the inspiratory capacity was calculated in relation to the body weight. After lung function testing at baseline level, mice were transferred to a Buxco R/C system (Troy, NY) to assess airway reactivity. Metacholine (MCh) was applied to the intubated mice via an in-line nebulizer and administered at increasing concentrations (0, 12.5, 25, 37.5 mg/ml) for 20 sec. Resistance (R) and Compliance (C) were measured continuously for 3 min. The average was calculated and plotted against the MCh concentration.

### Pediatric cohort

Peripheral blood mononuclear cells (PBMCs) were isolated from a subsample of 23 healthy children (age 4–15 years) with a history of early-life smoke exposure and compared to age-matched controls participating in the Munich Clinical Asthma Research Association (CLARA) study^32^. Early-life smoke exposure was defined as smoking of the mother during pregnancy and smoking of one or two parents at home at the time of inclusion into the study. ‘Non-smoking’ was defined as no smoking of either parent as well as no smoking at home throughout the pregnancy. According to this definition, six children were exposed (during pregnancy and currently at home), and 17 children had non-smoking parents. PBMC, isolated within 24 hours after blood withdrawal, were cultured (X-Vivo (Lonza, Basel, Switzerland) with plate-bound anti-CD3 (3 mg/mL) and soluble anti-CD28 (1 mg/mL) at 37 °C for 48 h. RNA was isolated with the RNeasy Mini-Kit and processed (1 mg) with reverse transcriptase (Qiagen, Hilden, Germany). Gene-specific PCR products of *IGF1* and *IGFBP3* were amplified with the CFX96 Touch Real-time-PCR Detection System (Bio-Rad, Munich, Germany) for 40 cycles.

### Ethics statement

The study has been approved by the local ethics committee of LMU Munich (project nr 379-08). All methods were carried out in accordance with relevant guidelines and regulations. Parents have agreed to participation in the study, and informed consent was obtained from all families.

### Gene expression analysis in murine lungs at embryonic day 18.5

#### Selection criteria of samples for RNA profiling

Both androgens and estrogens can influence lung development and physiology in prenatal life^33^. To limit undefined effects from interactions between neighboring siblings of different sexes, we selected fetuses which had two neighbors of identical sex or were positioned at the end of the uterus having a neighbor of the same sex (Supplementary Fig. S1). We further took one male and one female offspring per litter. In order to avoid detecting acute but transient effects, the mRNA and miRNA profiling were performed 24 h after the last cigarette smoke exposure (ED 18.5).

#### Expression profiling

Total RNA (n = 12/group; each six males and females) was isolated using the miRNeasy Mini (QIAGEN) kit. The Agilent 2100 Bioanalyzer was used to assess RNA quality where only high quality RNA, (RIN ≥ 8.7, 260/280 ratio > 1.8, no degradation as detected by RNA agarose gel), was used for microarray analysis. For mRNA profiling, 30 ng of total RNA was amplified using the Ovation PicoSL WTA System V2 in combination with the Encore Biotin Module (Nugen). Amplified cDNA was hybridized on an Affymetrix Mouse Gene ST 2.1 array plate. Staining and scanning were done according to the Affymetrix expression protocol including minor modifications as suggested in the Encore Biotin protocol. For microRNA profiling, 800 ng of total RNA was labeled with the FlashTag Biotin HSR kit (Genisphere) and hybridized on Affymetrix miRNA 3.0 arrays. Staining and scanning were done according to the Affymetrix expression protocol (Fluidics FS450_0002). Array data has been submitted to the GEO database at NCBI (GSE67888).

### Data analysis

#### Statistical transcriptome analysis for mRNA and microRNA profiling from mice

The expression console (v.1.3.0.187, Affymetrix) was used for quality control and to obtain annotated normalized RMA gene-level data and DABG (standard settings including median polish and sketch-quantile normalization). Three samples from the filtered-air group (one male, two females) haven’t fulfilled the quality criteria and were therefore excluded from further analysis. Redundant probe sets with identical expression values in all samples were removed from the dataset. Statistical analyses were performed using the statistical programming environment^34^ implemented in CARMAweb^35^. Gene wise testing for differential expression was done employing the (limma) *t*-test and Benjamini-Hochberg multiple testing correction (FDR < 10%). Sets of significantly regulated miRNAs were filtered for present calls in at least 60% of the samples in at least one group.

#### Upstream regulator and functional network analysis

The upstream regulator, network and functional analyses were generated through the use of IPA® (Ingenuity Pathway Analysis (QIAGEN)). First, the core analysis module of IPA® was applied to a set of 1340 CS-regulated (|FC| > 1.2, unadj. p < 0.05) mRNAs using default settings including direct and indirect relationships as well as endogenous metabolites. The Mouse Gene 2.1 ST Array was chosen as reference set. Molecules identified as upstream regulators of the dataset were filtered to those transcription regulators, which were regulated in the initial dataset. These molecules were displayed as a network together with their downstream targets. The “grow” function of IPA® was applied to the transcription regulators to identify CS-regulated microRNAs targeting these transcription regulators. Targeting microRNAs with non-inverse regulation were filtered out. An additional core analysis of the molecules in the upstream regulator network was used to identify the functional role of the top networks of these molecules.

### Statistical analyses

The weight gain of mothers and offspring was analyzed using SAS PROCs GLM and MIXED for repeated measure analyses (SAS Institute Inc. 2011. SAS® 9.3 System Options: Reference, Second Edition. Cary, NC: SAS Institute Inc.). Analyzing the weight gain of animals over time requires consideration in between-group effects as well as within subject effects. Typically, control and exposed groups started off with approximately equal weights but ended up being different in their average weight by the end of the study period. In general, the trend parameters, i.e. the shapes of the weight gain curves, differ between groups. This is accounted for by appropriately tested and chosen study group specific trend parameter interactions with time, e.g. by forward or backward selection. Weight gain and group* time interaction parameters were quantified by the natural measure milligram per day (mg/d) with 95%-confidence intervals (CI) and p-values. The corresponding synoptic graphical display of growth curves for control and exposure groups was obtained employing SAS PROC GPLOT. The remaining molecular data obtained in mice were tested for normal distribution using D’Agostino and Pearson omnibus normality test (GraphPAD Prism, GraphPad Software Inc. 2007). Mann-Whitney *U* test or t-test was applied where appropriate. Differences in mRNA expression levels of children with smoking and non-smoking parents were tested on log ∆CT scale using tobit regression analysis^36^.

### Data availability statement

The microarray data has been submitted to the GEO database at NCBI (GSE67888) and will be available for all readers upon publication.

## Results

### Effects of CS exposure on maternal characteristics of successful pregnancy

**Figure 1:**
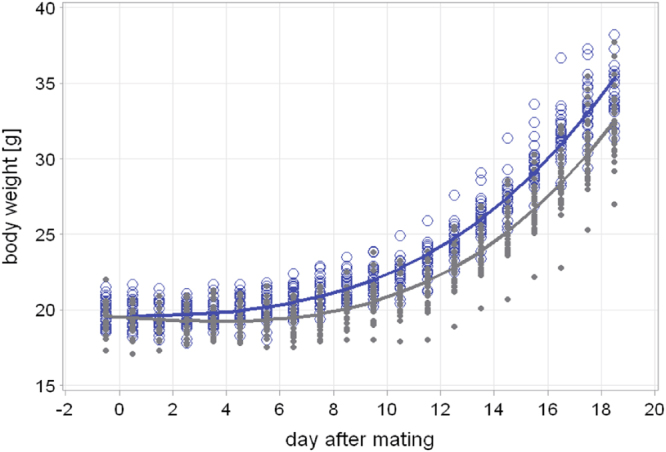
Weight trends of pregnant mice exposed either to CS or AIR. Dams were exposed daily to mainstream CS (grey) or AIR (blue) from ED 2.5 until delivery. Graphs depicts all observed values, especially the min and max values for each time point (open circles), and the corresponding optimum polynomial model fits (lines); p < 0.0001 after considering weight gain and group* time interactions and litter size. The interaction of litter size with smoke exposure was not significant and was therefore not included in the model.

Exposure to CS was monitored by assessing levels of urinary cotinine and peripheral blood CO-Hb, which were both significantly increased in CS-exposed female mice (Table 1). The average TPM exposure was 321 + 89 mg/m³. Eight-week old animals, as used in this study, still gain weight with the age. Age-related weight gain was clearly compromised in CS-exposed non-pregnant mice (mean −80 mg/d; CI 75–84 mg/d; p < 0.0001 Supplementary Fig. S2). This was also true for pregnant mice: CS-exposed dams lagged behind in weight gain by −157 mg/d (95%-CI: 145–168), p < 0.0001 (Fig. 1). This difference remained significant even after the maternal weight was corrected for the size of the litter. Despite the effect of CS on maternal weight gain, the pregnancy rate, duration and number of viable pups or visibly resorbed fetuses did not differ significantly between the groups. Similarly, the number of female and male offspring were equal in both groups indicating that the parameters of successful pregnancy *per se* were not affected by the CS protocol applied (Table 1).

**Table 1:** Characteristics of maternal exposure and reproduction.

	AIR	CS	p-value
Urine cotinine [mg/ml]#* (n)	117 ± 26 7	600 ± 25 15	<0.0001
Co-Hb [%] #* (n)	1.3 ± 0.2 17	6.3 ± 0.6 22	<0.0001
Pregnancy rate [%] (n)	13.1 ± 8.8 32	9.6 ± 6.9 29	0.3
Pregnancy duration [d] (n)	19.8 ± 0.5 12	19.8 ± 0.6 12	>0.99
Litter size (n)	6.2 ± 1.8 32	5.7 ± 1.9 29	>0.99
Visible resorptions (n)	0.5 ± 0.6 20	1.0 ± 0.5 17	0.1
Male offspring [%] (n)	43.2 ± 10.2 77	51.3 ± 23.0 72	0.3
Maternal weight at section [g] (n)	34.4 ± 2.1 15	32.8 ± 2.4 8	0.1

^#^Mean ± SD, *analyzed in non-pregnant animals; Pregnancy rate: percentage of female mice with successfully established pregnancy after overnight 1:1 mating.

### Reduced intrauterine and postnatal growth of exposed offspring

**Figure 2:**
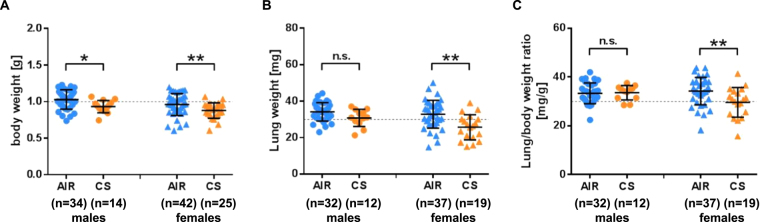
Intrauterine growth retardation after CS exposure. Weight at cesarean section (E18.5) (**A**) body weight (**B**), lung weight and (**C**), lung-to-body weight ratios of fetuses. AIR (blue) or CS-exposed (orange) male (•) and females (▲) fetuses. Litter size > 6, mean ± SD; unpaired *t* test (*p < 0.05, **p < 0.01, ***p < 0.001, ****p < 0.0001).

**Figure 3:**
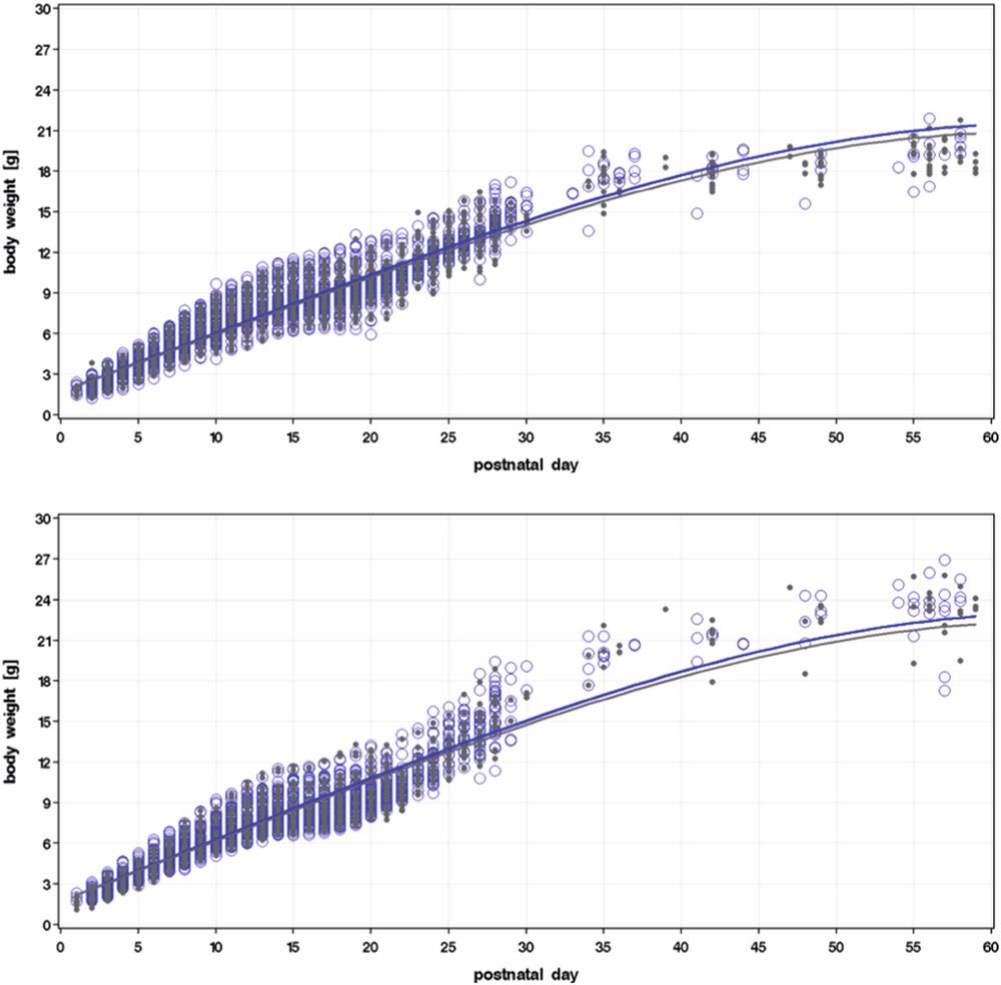
Intrauterine smoke exposure reduces postnatal growth weight gain. Postnatal weight gain of female (upper panel) and male (lower panel) offspring until PND 56 after i*n utero* CS (grey) or AIR (blue) exposure. Graphs depicts all observed values, especially the min and max values for each time point (open circles), and the corresponding optimum polynomial model fits (lines); p < 0.0001 after considering weight gain and group* time interactions.

Smoke-exposed fetuses (ED18.5) had significantly lower body and lung weights as compared to the air-exposed animals. Pulmonary hypotrophy, as indicated by lower lung/body weight ratio, was more pronounced in female offspring resulting in lower lung/body weight ratios in females but not in males (Fig. 2A–C). As expected, postnatal weight gain was lower in females independent of prenatal CS-exposure. Furthermore, the offspring from litters with less than six animals had a stronger weight gain as compared to animals from larger litters (>6) possibly due to the different breast milk availability per animal. Prenatal CS-exposure retarded weight gain in offspring as investigated until PND 56 (Fig. 3A,B). When we corrected our analyses for sex and litter size, the reduced postnatal weight gain of prenatally CS-exposed animals remained highly significant (mean −6.7 mg/d, 95%-CI [4.0, 9.4]).

#### Reduced lung function in offspring

**Figure 4:**
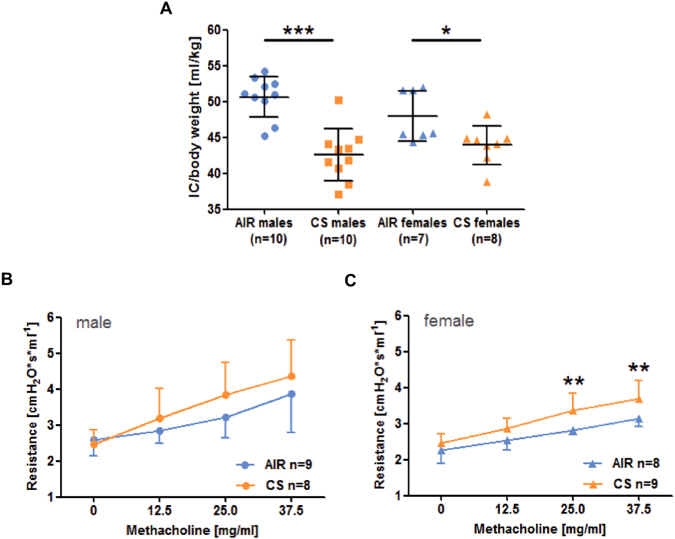
*In utero* CS exposure affects lung function in adolescent offspring. (**A**) Inspiratory Capacity (IC)/body weight; (**B**,**C**), Airway hyperreactivity (AHR); Male (•) and female (▲) offspring; measured at d21 after birth; AIR- (blue), CS-exposure (orange). mean ± SD, MWU; (*p < 0.05; **p < 0.01 ***; p < 0.001).

Next, we investigated whether smoke-induced intrauterine and postnatal growth retardation was associated with lung function deficits. We found that the inspiratory capacity was significantly reduced in three-week old animals after intrauterine CS-exposure. When we stratified for sex, this decrease was present in both males and females (Fig. 4A). In contrast, airway hyperreactivity was only seen in female offspring, even in the absence of any experimental allergen challenge (Fig. 4B,C).

#### Gene expression networks in fetal lungs

With the establishment of our model that mimics findings of prenatally smoke exposed children, we next aimed to shed more light on the molecular mechanisms preceding impaired lung function of CS-exposed offspring. Therefore, we performed mRNA and microRNA arrays in fetal lungs, which revealed 1340 differentially regulated mRNAs (|FC| > 1.2, unadj. p-value < 0.05) and 133 microRNAs (unadj. p-value < 0.05) (Supplementary Fig. S3). The array data is also available at the GEO database at NCBI (GSE67888).

**Figure 5:**
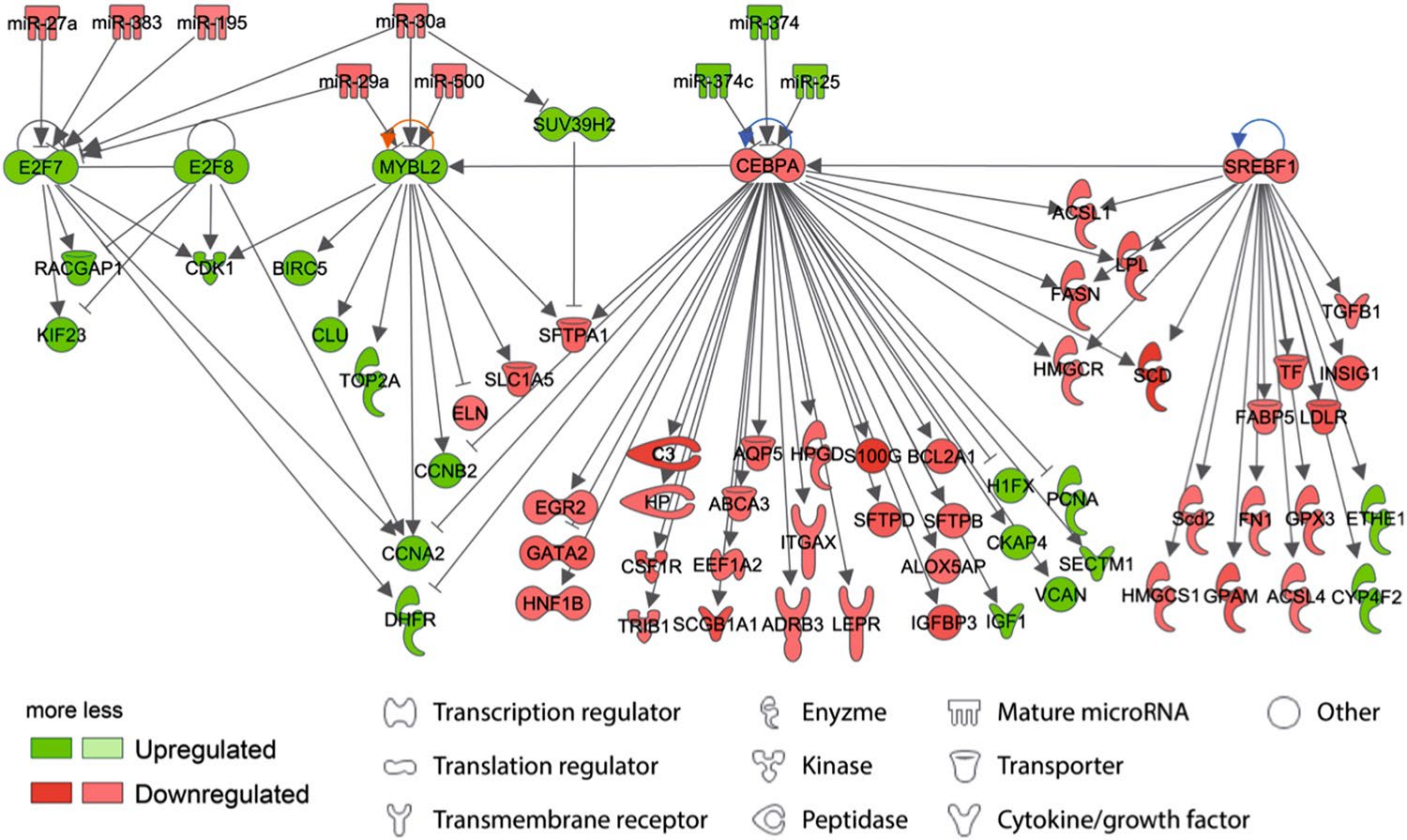
Network of top deregulated transcriptional regulatory factors in fetal smoke exposed lungs (E18.5). The core analysis module of the Ingenuity® software was used to identify top biological functions of 1340 mRNAs (Affymetrix Mouse Gene ST 2.1 array) deregulated by CS in fetal lungs (female and male merged). This dataset was filtered to transcriptional regulators showing deregulation and are displayed as a network together with their downstream targets.

**Figure 6:**
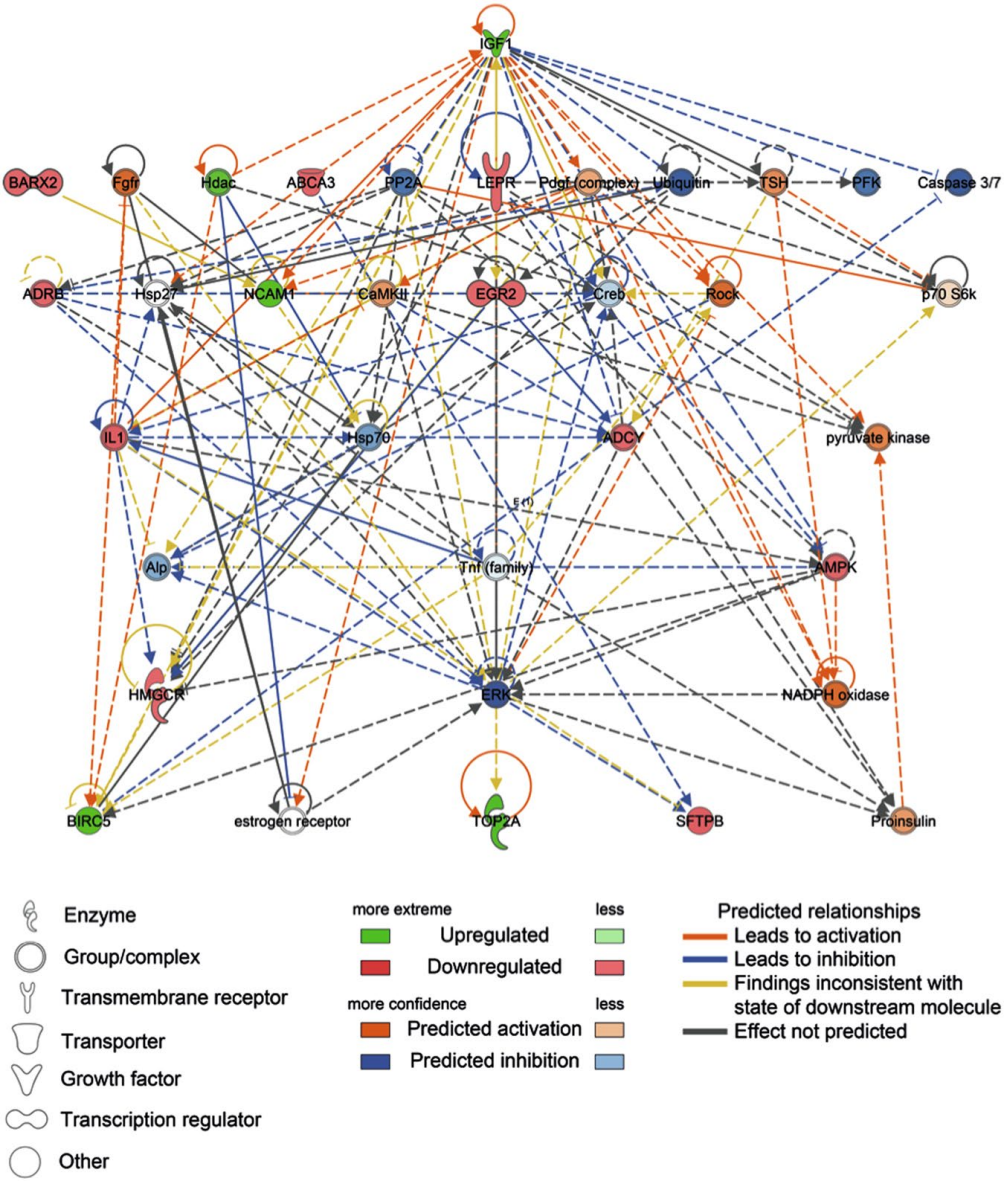
*Igf1* is the top hierarchical node of transcriptional regulators after intrauterine CS exposure Counter-regulated microRNAs targeting the network of transcriptional regulators and their downstream targets were identified and included in this dataset. (E18.5, female and male merged). To identify the functional role of the top networks of these molecules, a second core analysis of the molecules of this enlarged dataset was applied. The results are visualized as network.

To identify top biological functions associated with changes in global mRNA expression patterns, the core analysis module of the Ingenuity® software was used. Besides other functions (Supplementary Table S2), those associated with embryonic and organismal development were strongly affected by *in utero* smoking (Supplementary Table S3). To investigate which key developmental molecules could be responsible for these changes, we identified upstream regulators within the dataset and filtered those to transcription regulators which showed mRNA dysregulation. These molecules were displayed as a network together with their downstream targets (Fig. 5). Three transcription factors involved in transcriptional repression such as E2F transcription factors 7 and 8 (E2f7, E2f8^37^), Myb-related protein B (Mybl2)^38^ responsible for control of cellular proliferation and senescence and histone-lysine N-methyltransferase (Suv39h2) which is highly specific for H3K9 trimethylation^39^, were up-regulated. In contrast, two transcription factors related to lipid metabolism (CCAAT/enhancer binding protein (C/EBP) alpha (Cebpa) and Sterol regulatory element binding transcription factor 1 (Srebf1)) were down-regulated. In a next step, we included counter-regulated miRNAs targeting the transcription factors to the network, as miRNAs are an additional important level of regulation. Most down-regulated functions involved lipid metabolism and survival, whereas the top up-regulated functions were related to inflammatory and respiratory diseases (Supplementary Table S4). To gain insight into the interactions of these molecules, we performed a network analysis and found Insulin-like growth factor 1 (Igf1) as top hierarchical node in the subnetwork associated with embryonic and organ development (Fig. 6). The *in silico* analysis of this Igf1-driven subnetwork revealed organismal survival, organismal injury and abnormalities and, most importantly, respiratory disease as its major function (Table 2).

**Table 2:** Top 20 Diseases and Biofunctions associated with changes in IGF1 network mRNA expression patterns (diseases and functions significant to network molecules).

Categories	Diseases or Functions Annotation	p-Value	# Molecules
Organismal Survival	organismal death	3.64E-05	15
	survival of organism	3.90E-05	11
Organismal Injury & Abnormalities	lymphocytic cancer	1.11E-05	9
	non-Hodgkin’s disease	6.94E-05	8
	biliary tract tumor	6.71E-05	4
	biliary tract cancer	6.71E-05	3
Respiratory Disease	obstructive pulmonary disease	7.95E-06	5
	atelectasis	2.89E-05	3
	small cell lung cancer	6.32E-05	3
	abnormal composition of pulmonary surfactant	2.60E-06	2
	congenital pulmonary alveolar proteinosis	3.66E-06	2
	community acquired pneumonia	1.06E-05	2
	abnormal secretion of pulmonary surfactant	4.90E-05	2
	primary atelectasis	9.67E-05	2
Cell Death & Survival	apoptosis of myeloma cell lines	5.45E-05	5
	survival of hepatic stellate cells	2.87E-05	2
DNA Replication. Recombination & Repair	DNA damage	3.18E-05	5
Respiratory System Development & Function	abnormal morphology of type II pneumocytes	1.11E-06	3
Inflammatory Response	cytotoxic reaction of breast cancer cell lines	3.03E-05	2
Organ Development	growth of mammary duct	6.59E-05	2

Some diseases and functions appear in more than one category. Only one category for each function is shown for simplicity. *The p-value of overlap was calculated by the Fisher’s Exact Test.

### Pulmonary Igf1 and Igf-binding protein (Igfbp3) expressions in mice and exposed children

**Figure 7:**
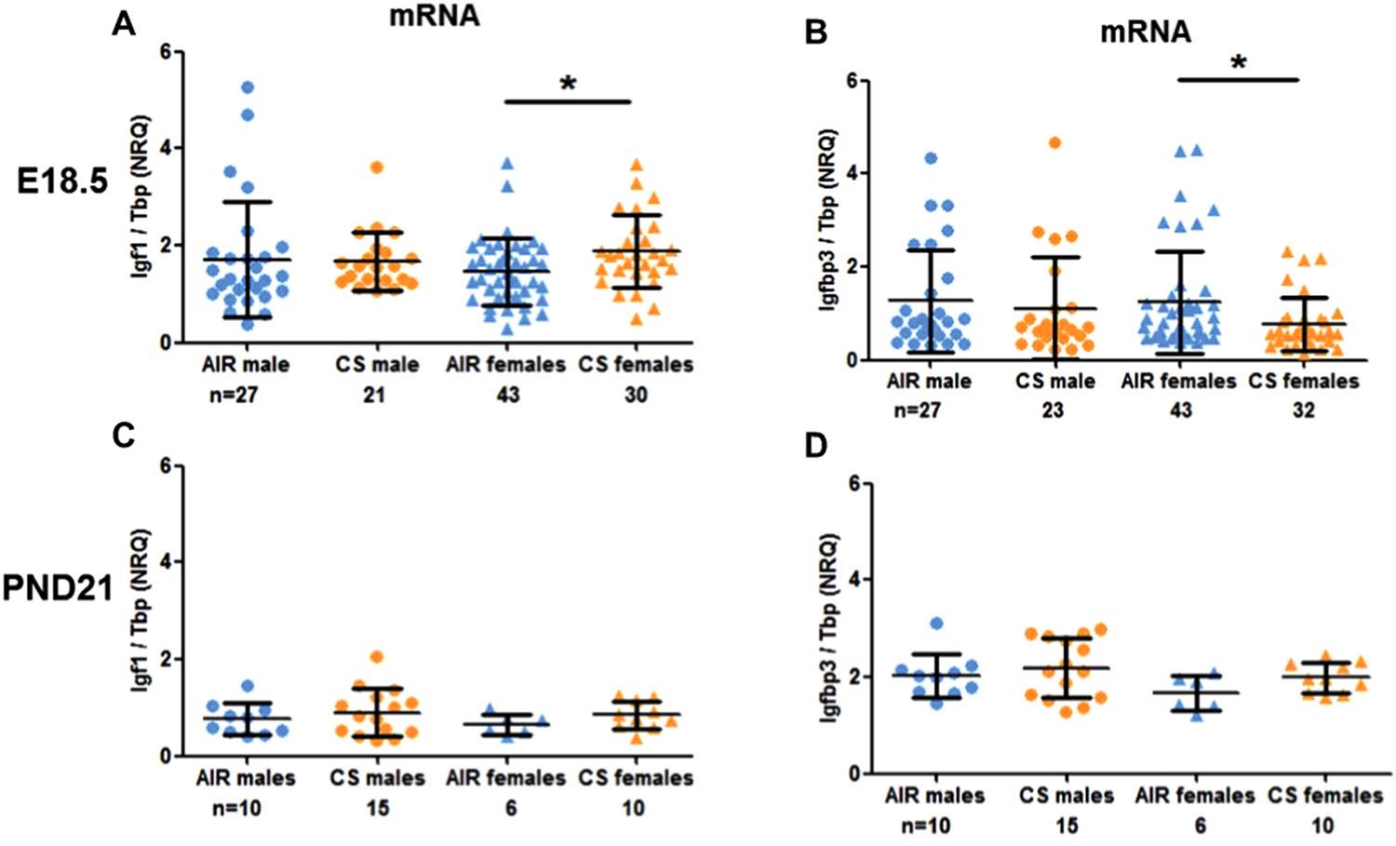
Pulmonary gene expressions of *Igf1* and *Igfbp3* after intrauterine CS exposure. *Igf1* (**A**,**C**) and *Igfbp3* (**B**,**D**) gene expression in lungs of E18.5 and PND21 males (•) and females (▲); AIR- (blue) and CS- (orange) exposure. Normalized relative quantity (NRQ) with TATA-binding protein (Tbp) as reference gene. Mean + SD, n ≥ 6; MWU (*p < 0.05).

**Figure 8:**
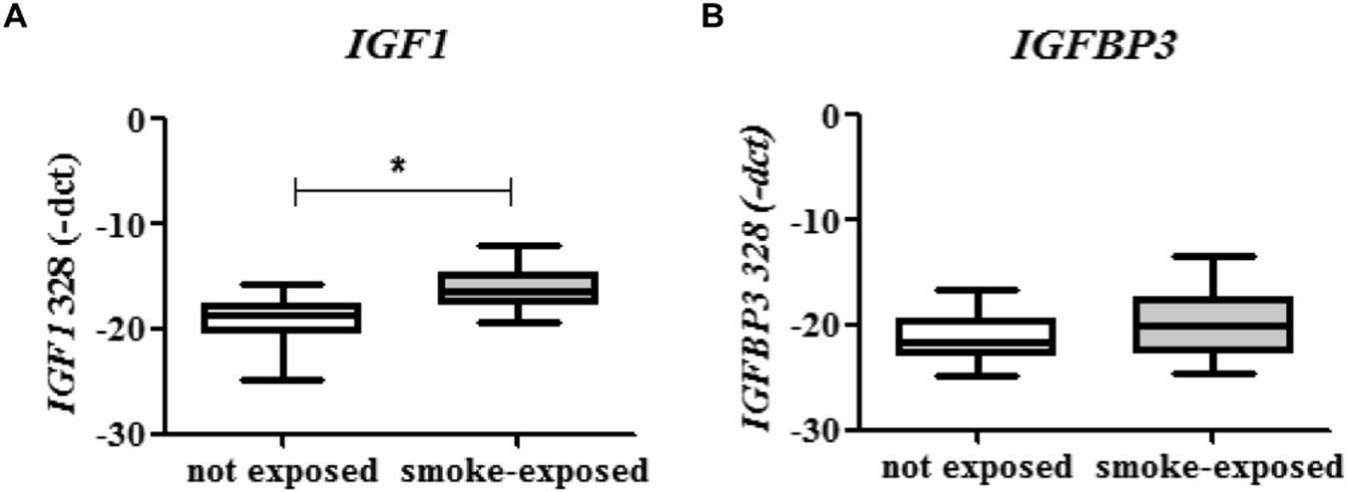
*IGF1* is increased in PBMCs of early life smoke-exposed children. mRNA expression of *IGF1* (**A**) and *IGFBP3* (**B**) following anti-CD3/CD28-stimulation.; p-value from Tobit regression analysis (median ± IQR); n(smoking parents) = 6, n(non-smoking parents) = 17. −∆CT (target – reference gene) values are shown in boxplots, indicating median, quartiles and min/max values. Higher −∆CT indicates higher expression. (*p < 0.05).

Since the *in silico* analyses suggested Igf1 as a main driver of reduced lung weight and function, we further focused on this growth hormone. Igf1 is almost completely bound to one of the six binding proteins (Igfbp). Igfbp3 is the most abundant protein that accounts for the majority of all IGF binding. As it is also downregulated in our array (Fig. 5) we additionally included this hormone in the analysis. *Igf1* mRNA expression was significantly increased in female prenatally smoke-exposed lungs at day E18.5, whereas *Igfbp3* expression wa*s* decreased. This effect was no longer found in offspring of three weeks of age (Fig. 7). To determine whether our model more or less reflects the human situation, we investigated anti-CD3/CD28-stimulated PBMCs of healthy school-aged children with early-life smoke exposure. Interestingly, mRNA expression of *IGF1* was significantly higher in PBMCs from children with smoking parents as compared to children from non-smoking parents, whereas mRNA expression of *IGFBP3* was not affected (Fig. 8, p = 0.008; p = 0.16).

## Discussion

In this study, we aimed to investigate underlying molecular mechanisms for lung development deficits due to *in utero* cigarette smoke exposure by using an animal model of maternal smoking during pregnancy. Prenatally exposed mice showed intrauterine and postnatal growth retardation and several alterations on the pulmonary transcriptomic and miRNA levels at E18.5. In addition to this, a reduction of lung function in adolescence was detected, which is of interest as it is a recognized smoking-induced pathology in children^3,4,40–43^. Extensive mRNA/miRNA profiling of fetal lungs revealed a dysregulation of 1340 mRNAs and 133 miRNAs of CS-exposed offspring compared to controls. A subsequent *in silico* construction of networks of transcriptional regulators, revealed, beyond others, *Igf1* as major hub in fetal smoke exposed lungs. Deregulation of pulmonary *Igf1* together with its main binding partner *Igfbp3* was also confirmed by qRT-PCR in murine offspring and further corroborated in school-aged children by showing reduced *IGF1* expression in PBMC after prenatal and early-life smoke exposure.

As our goal was to investigate the consequences that are purely related to prenatal smoke-exposure and not to a compromised pregnancy by maternal stress, we designed the exposure in a way that the basic parameters of successful pregnancy remained unaltered. As smoking is linked to reduced fertility and increased rates of abortion^44^, mice were subjected to smoke after fertilization but prior to the formation of the lung bud. In order to compare the severity of our smoke exposure to other studies and human data, we assessed cotinine, a nicotine metabolite in urine, and carboxyhemoglobin (Co-Hb) levels in the blood.

We observed an 5.12-fold increase of urine cotinine levels, and a 4.84-fold increase in Co-Hb in our model in smoke exposed mothers compared to air controls. However, it has to be noted that in this study samples were drawn 4 h after the last smoke exposure, and the half-time of cotinine in C57BL/6 mice is only 38 min^45^. Thus, we assume the fold-increase in urine cotinine levels are reflecting light smoking when compared to data from a large cohort of pregnant women^46^. Nonetheless, intrauterine growth retardation was pronounced and – more importantly - sustained into adulthood, while smoking was stopped at birth. Surprisingly, the offspring did not show catch-up weight gain after weaning, even after the adjustment for sex, litter size and consideration of seasonal effect; thus it is intriguing to speculate that intrauterine smoke-exposure causes a persistent developmental disadvantage.

We observed lung function deficits in CS-exposed offspring at the age of three weeks. Of note, female offspring exhibited spontaneous airway hyperreactivity (AHR), even in the absence of allergen challenge, and showed a significant higher expression of *Igf1* at E18.5, thus indicating a possible link between *Igf1* and lung function. It is intriguing to speculate that the observed lung function differences are due to increased airway remodeling, which has been previously reported in similar models of prenatal smoke exposure^47^, which we however could not investigate in this study.

Sexual dimorphism is well known in physiological and non-physiological lung responses^48^ including developmental, hormonal, airway structural and functional differences. For example, female fetuses start mouth movements^49^, surfactant synthesis and maturation earlier than male fetuses^50^. In early childhood, girls have larger airways in relation to lung size, and lower specific airway resistance than boys (reviewed in^51^). Thus, it is conceivable that environmental insults act differently on females and males during development. Nonetheless, the assessment of sex differences has so far been neglected in most inter- or trans- generational animal models.

Fetal smoke-exposed lungs showed regulation of signaling molecules at multiple levels from transcription regulators to their down-stream targets. To avoid seeing acute and transient effects, arrays were performed one day after the last smoke exposure on pups delivered by c-section (E18.5). Thus, even though the fold–changes of gene expression were low, they might indicate the onset of pathology and hence be more relevant than stronger events occurring at the end of molecular cascades.

Lung development is a precisely controlled process of parallel proliferation, differentiation and apoptosis. Transcriptional regulator network showed dysregulation of the genes that are relevant for development and the onset of respiratory diseases. The transcription factors *E2f7* and *E2f8* protect cooperatively against excessive apoptosis via repression of *E2f1* and are strictly required for embryonic development^37,52^. *Mybl2*, which controls cell cycle progression and cell fate in embryonic stem cells^2^, was equally upregulated implying disturbed early developmental processes. On the other hand, *Cebpa* and *Srbf1* which are considered to be critical for lung maturation and respiratory epithelial differentiation (*Cebpa*), were decreased^53–55^.

IGF1 is an important regulator of somatic growth and cell differentiation^56^ where the absence of its signaling results in severe growth failure^57,58^. A specific role of *IGF1* for lung development is indicated by strong *IGF1* upregulation in lung structural cells in bronchopulmonary dysplasia^59^ or severe pulmonary hypoplasia resulting from diaphragmatic hernia^60^. *Igf1* deficient mice were shown to exhibit growth failure which was more pronounced in lungs as compared to body weight^61,62^.

So far, data relating prenatal smoke exposure to changes of pulmonary IGF-1 are limited. One study in adult male smokers reported reduced serum IGF-1 and IGFBP-3 levels that returned to normal levels after smoking cessation^63^. Two mouse studies have previously implicated the *Igf1* axis with prenatal smoke exposure, and of note both found sex-specific effects as we did in this study. The first one used Balb/c mice that were nose-only exposed during the entire pregnancy, and was in contrast to our findings showing lower *Igf1* mRNA expression in 30-day old female offspring in comparison to control^29^. This was related to sex-dependent differences in promoter methylation of *Igf1r*. This was recently confirmed in a second study using a prenatal smoke model in C57Bl/6 mice, which also showed that Igfr1 CpG methylation was organ- and sex-specific^64^. The difference to our study could be due to the different time point of analysis as the observed increase in *Igf1* levels in our study was only found at E18.5, but not at PND 21 and we did not investigate d30. Along this line, it has been found that *Igf1* levels decrease postnatally^65^. Concerning the *Igf1* levels in male offspring, our results were in accordance to the abovementioned study by Meyer *et al*. showing no significant change in *Igf1* expression. However, other investigators did not reveal such a relationship^66^.

In humans, maternal smoking in pregnancy has been associated with lower placental GH and human cord blood IGF-1 protein concentrations^67^. Another study, as well, showed a lower cord blood IGF-1 in infants from mothers who smoked during early pregnancy, with the strongest decline in girls as compared to boys^68^. Similar to our findings in mice, the growth hormone deregulation was more pronounced in females. In this study, we partly validated our findings from the mouse model by the observation that *IGF-1* levels are increased in PBMCs from *in utero* CS-exposed, healthy children at school age. However, the number of human samples used in the present study did not allow stratifying for sex. Further, these children were healthy; thus we do not have any further information on whether they had subtle deficits in lung function and as lung development is vastly different between mice and humans^69^ they do not reflect the same developmental stages as in our murine model. Nonetheless, this is a first hint that *IGF-1* dysregulation might be an important effector of *in utero* CS-exposure and might indicate a certain ‘molecular defect’ in smoke-exposed children, which could for example result in a changed susceptibility to develop chronic lung diseases such as asthma in later life. Thus, this needs to be further investigated in future studies.

We acknowledge several potential limitations of our study. First, similar to human studies^2^, murine models do not allow to clearly separate the effects of intrauterine growth retardation from smoke exposure on the developing lung. Second, despite the apparently normal course of pregnancy, placental functions could be affected. Prenatal smoking, albeit at higher doses, has been shown to influence decidua immune responses in mice and humans^70^. Nicotine exposure has further been linked to altered placental development and function in rats^71^. In humans, maternal smoking has been related to global^72^ and gene-specific^73^ placental DNA-methylation thus indicating an abnormal placental function. Third, although air and smoke test animals were handled in the same way, smoking, is an additional – although unavoidable -, stress factor itself. Moreover, maternal stress during pregnancy can induce low-level maternal corticosterone^74^ and increase responsiveness to allergen challenge in offspring^74,75^. Thus, it is possible that besides smoking, elevated maternal stress hormones might have contributed to our findings.

In summary, we here show that prenatal smoke exposure is associated with deregulated pulmonary transcriptomes, miRNA levels and *Igf1* in a sex-specific manner. To our knowledge we are the first to report a change in pulmonary transcriptomes and miRNA levels at day E18.5, which Ingenuity Pathway Analysis (IPA) predicted to be involved in respiratory disease as one of the top hits. These findings, taken together with the previously reported Igf1 dysregulation might not only indicate an aberrant lung development, but might also alter the offsprings’ response to environmental insults, such as allergens or cigarette smoke and therewith predispose for the development of chronic lung diseases.

## Electronic supplementary material

Supplementary Information

## References

[1] Benjamin-Garner R, Stotts A (2013). Impact of smoking exposure change on infant birth weight among a cohort of women in a prenatal smoking cessation study. Nicotine Tob Res.

[2] Bjerg A, Hedman L, Perzanowski M, Lundback B, Ronmark E (2011). A strong synergism of low birth weight and prenatal smoking on asthma in schoolchildren. Pediatrics.

[3] Moshammer H (2006). Parental smoking and lung function in children: an international study. Am J Respir Crit Care Med.

[4] Hollams EM, de Klerk NH, Holt PG, Sly PD (2014). Persistent effects of maternal smoking during pregnancy on lung function and asthma in adolescents. Am J Respir Crit Care Med.

[5] Gilliland FD, Berhane K, Li YF, Rappaport EB, Peters JM (2003). Effects of early onset asthma and in utero exposure to maternal smoking on childhood lung function. Am J Respir Crit Care Med.

[6] Maritz GS, Harding R (2011). Life-long programming implications of exposure to tobacco smoking and nicotine before and soon after birth: evidence for altered lung development. Int J Environ Res Public Health.

[7] Duijts L (2012). Fetal exposure to maternal and paternal smoking and the risks of wheezing in preschool children: the Generation R Study. Chest.

[8] Burke H (2012). Prenatal and passive smoke exposure and incidence of asthma and wheeze: systematic review and meta-analysis. Pediatrics.

[9] den Dekker HT (2015). Tobacco Smoke Exposure, Airway Resistance, and Asthma in School-age Children: The Generation R Study. Chest.

[10] Beyer D, Mitfessel H, Gillissen A (2009). Maternal smoking promotes chronic obstructive lung disease in the offspring as adults. Eur J Med Res.

[11] Svanes C (2010). Early life origins of chronic obstructive pulmonary disease. Thorax.

[12] Duijts L, Reiss IK, Brusselle G, de Jongste JC (2014). Early origins of chronic obstructive lung diseases across the life course. Eur J Epidemiol.

[13] Leone FT (2015). An Official American Thoracic Society Research Statement: Current Understanding and Future Research Needs in Tobacco Control and Treatment. Am J Respir Crit Care Med.

[14] Golding J, Northstone K, Gregory S, Miller LL, Pembrey M (2014). The anthropometry of children and adolescents may be influenced by the prenatal smoking habits of their grandmothers: a longitudinal cohort study. Am J Hum Biol.

[15] Miller LL, Pembrey M, Davey Smith G, Northstone K, Golding J (2014). Is the growth of the fetus of a non-smoking mother influenced by the smoking of either grandmother while pregnant?. PLoS One.

[16] Li YF, Langholz B, Salam MT, Gilliland FD (2005). Maternal and grandmaternal smoking patterns are associated with early childhood asthma. Chest.

[17] Magnus MC (2015). Grandmother’s smoking when pregnant with the mother and asthma in the grandchild: the Norwegian Mother and Child Cohort Study. Thorax.

[18] Miller LL, Henderson J, Northstone K, Pembrey M, Golding J (2014). Do grandmaternal smoking patterns influence the etiology of childhood asthma?. Chest.

[19] Rehan VK, Liu J, Sakurai R, Torday JS (2013). Perinatal nicotine-induced transgenerational asthma. Am J Physiol Lung Cell Mol Physiol.

[20] Raaberg L (1992). Epidermal growth factor transcription, translation, and signal transduction by rat type II pneumocytes in culture. Am J Respir Cell Mol Biol.

[21] Raaberg L, Nexo E, Poulsen SS, Jorgensen PE (1995). An immunologic approach to induction of epidermal growth factor deficiency: induction and characterization of autoantibodies to epidermal growth factor in rats. Pediatr Res.

[22] Raaberg L, Nexo E, Jorgensen PE, Poulsen SS, Jakab M (1995). Fetal effects of epidermal growth factor deficiency induced in rats by autoantibodies against epidermal growth factor. Pediatr Res.

[23] Warburton D (1992). Epigenetic role of epidermal growth factor expression and signalling in embryonic mouse lung morphogenesis. Dev Biol.

[24] Minoo P, King RJ (1994). Epithelial-mesenchymal interactions in lung development. Annu Rev Physiol.

[25] Peters K (1994). Targeted expression of a dominant negative FGF receptor blocks branching morphogenesis and epithelial differentiation of the mouse lung. EMBO J.

[26] Kaartinen V (1995). Abnormal lung development and cleft palate in mice lacking TGF-beta 3 indicates defects of epithelial-mesenchymal interaction. Nat Genet.

[27] Epaud R (2012). Knockout of insulin-like growth factor-1 receptor impairs distal lung morphogenesis. PLoS One.

[28] Liu JP, Baker J, Perkins AS, Robertson EJ, Efstratiadis A (1993). Mice carrying null mutations of the genes encoding insulin-like growth factor I (Igf-1) and type 1 IGFreceptor (Igf1r). Cell.

[29] Meyer KF (2017). Prenatal exposure to tobacco smoke sex dependently influences methylation and mRNA levels of the Igf axis in lungs of mouse offspring. Am J Physiol Lung Cell Mol Physiol.

[30] Clifton VL (2010). Effect of maternal asthma, inhaled glucocorticoids and cigarette use during pregnancy on the newborn insulin-like growth factor axis. Growth Horm IGF Res.

[31] John G (2014). The composition of cigarette smoke determines inflammatory cell recruitment to the lung in COPD mouse models. Clin Sci (Lond).

[32] Raedler D (2015). Identification of novel immune phenotypes for allergic and nonallergic childhood asthma. J Allergy Clin Immunol.

[33] Melgert BN, Ray A, Hylkema MN, Timens W, Postma DS (2007). Are there reasons why adult asthma is more common in females?. Curr Allergy Asthma Rep.

[34] R Development Core Team. R: A language and environment for statistical computing. (R Foundation for Statistical Computing Vienna, Austria. http://www.R-project.org/), 2011.

[35] Rainer J, Sanchez-Cabo F, Stocker G, Sturn A, Trajanoski Z (2006). CARMAweb: comprehensive R- and bioconductor-based web service for microarray data analysis. Nucleic Acids Res.

[36] Ballenberger N, Lluis A, von Mutius E, Illi S, Schaub B (2012). Novel statistical approaches for non-normal censored immunological data: analysis of cytokine and gene expression data. PLoS One.

[37] Li J (2008). Synergistic function of E2F7 and E2F8 is essential for cell survival and embryonic development. Dev Cell.

[38] Mowla SN, Lam EW, Jat PS (2014). Cellular senescence and aging: the role of B-MYB. Aging Cell.

[39] Schuhmacher MK, Kudithipudi S, Kusevic D, Weirich S, Jeltsch A (2015). Activity and specificity of the human SUV39H2 protein lysine methyltransferase. Biochim Biophys Acta.

[40] Martinez-Mesa J (2012). Life course association of maternal smoking during pregnancy and offspring’s height: data from the 1993 Pelotas (Brazil) birth cohort. J Adolesc Health.

[41] Iniguez C (2013). Maternal smoking during pregnancy and fetal biometry: the INMA Mother and Child Cohort Study. Am J Epidemiol.

[42] Delpisheh A, Brabin L, Drummond S, Brabin BJ (2008). Prenatal smoking exposure and asymmetric fetal growth restriction. Ann Hum Biol.

[43] Horta BL, Victora CG, Menezes AM, Halpern R, Barros FC (1997). Low birthweight, preterm births and intrauterine growth retardation in relation to maternal smoking. Paediatr Perinat Epidemiol.

[44] Castles A, Adams EK, Melvin CL, Kelsch C, Boulton ML (1999). Effects of smoking during pregnancy. Five meta-analyses. Am J Prev Med.

[45] Siu EC, Tyndale RF (2007). Characterization and comparison of nicotine and cotinine metabolism *in vitro* and *in vivo* in DBA/2 and C57BL/6 mice. Mol Pharmacol.

[46] Pickett KE, Rathouz PJ, Kasza K, Wakschlag LS, Wright R (2005). Self-reported smoking, cotinine levels, and patterns of smoking in pregnancy. Paediatr Perinat Epidemiol.

[47] Blacquiere MJ (2009). Maternal smoking during pregnancy induces airway remodelling in mice offspring. Eur Respir J.

[48] Bjerg A (2013). Higher risk of wheeze in female than male smokers. Results from the Swedish GA 2 LEN study. PLoS One.

[49] Hepper PG, Shannon EA, Dornan JC (1997). Sex differences in fetal mouth movements. Lancet.

[50] Torday JS, Nielsen HC (1987). The sex difference in fetal lung surfactant production. Exp Lung Res.

[51] Becklake MR, Kauffmann F (1999). Gender differences in airway behaviour over the human life span. Thorax.

[52] Ouseph MM (2013). Atypical E2F repressors and activators coordinate placental development. Dev Cell.

[53] Xu Y, Ikegami M, Wang Y, Matsuzaki Y, Whitsett JA (2007). Gene expression and biological processes influenced by deletion of Stat3 in pulmonary type II epithelial cells. BMC Genomics.

[54] Sato A, Xu Y, Whitsett JA, Ikegami M (2012). CCAAT/enhancer binding protein-alpha regulates the protease/antiprotease balance required for bronchiolar epithelium regeneration. Am J Respir Cell Mol Biol.

[55] Xu Y (2012). Transcriptional programs controlling perinatal lung maturation. PLoS One.

[56] Lopez IP (2015). Differential organ phenotypes after postnatal Igf1r gene conditional deletion induced by tamoxifen in UBC-CreERT2; Igf1r fl/fl double transgenic mice. Transgenic Res.

[57] Guevara-Aguirre J (2011). Growth hormone receptor deficiency is associated with a major reduction in pro-aging signaling, cancer, and diabetes in humans. Sci Transl Med.

[58] Kruis T (2010). Heterozygous mutation within a kinase-conserved motif of the insulin-like growth factor I receptor causes intrauterine and postnatal growth retardation. J Clin Endocrinol Metab.

[59] Chetty A, Andersson S, Lassus P, Nielsen HC (2004). Insulin-like growth factor-1 (IGF-1) and IGF-1 receptor (IGF-1R) expression in human lung in RDS and BPD. Pediatr Pulmonol.

[60] Miyazaki E, Ohshiro K, Taira Y, Puri P (1998). Altered insulin-like growth factor I mRNA expression in human hypoplastic lung in congenital diaphragmatic hernia. J Pediatr Surg.

[61] Wang J, Zhou J, Powell-Braxton L, Bondy C (1999). Effects of Igf1 gene deletion on postnatal growth patterns. Endocrinology.

[62] Pais RS (2013). Transcriptome analysis in prenatal IGF1-deficient mice identifies molecular pathways and target genes involved in distal lung differentiation. PLoS One.

[63] Renehan AG, Atkin WS, O’Dwyer ST, Shalet SM (2004). The effect of cigarette smoking use and cessation on serum insulin-like growth factors. Br J Cancer.

[64] Meyer KF (2017). The fetal programming effect of prenatal smoking on Igf1r and Igf1 methylation is organ- and sex-specific. Epigenetics.

[65] Copeland KC, Colletti RB, Devlin JT, McAuliffe TL (1990). The relationship between insulin-like growth factor-I, adiposity, and aging. Metabolism.

[66] Palmer RM, Wilson RF, Coward PY, Scott DA (2002). Analysis of circulating insulin-like growth factor-1 (IGF-1) and IGF binding protein-3 (IGFBP-3) in tobacco smokers and non-smokers. Tob Induc Dis.

[67] Coutant R (2001). Relationships between placental GH concentration and maternal smoking, newborn gender, and maternal leptin: possible implications for birth weight. J Clin Endocrinol Metab.

[68] Fleisch AF (2017). Associations of maternal prenatal smoking with umbilical cord blood hormones: the Project Viva cohort. Metabolism.

[69] Metzger RJ, Klein OD, Martin GR, Krasnow MA (2008). The branching programme of mouse lung development. Nature.

[70] Prins JR (2012). Smoking during pregnancy influences the maternal immune response in mice and humans. Am J Obstet Gynecol.

[71] Zhang H (2014). The interplay of DNA methylation over time with Th2 pathway genetic variants on asthma risk and temporal asthma transition. Clin Epigenetics.

[72] Suter M (2011). Maternal tobacco use modestly alters correlated epigenome-wide placental DNA methylation and gene expression. Epigenetics.

[73] Suter M (2010). In utero tobacco exposure epigenetically modifies placental CYP1A1 expression. Metabolism.

[74] Lim R, Fedulov AV, Kobzik L (2014). Maternal stress during pregnancy increases neonatal allergy susceptibility: role of glucocorticoids. Am J Physiol Lung Cell Mol Physiol.

[75] Pincus-Knackstedt MK (2006). Prenatal stress enhances susceptibility of murine adult offspring toward airway inflammation. J Immunol.

