# Supplementary material for: Intrauterine smoke exposure deregulates lung function, pulmonary transcriptomes, and in particular insulin-like growth factor (IGF)-1 in a sex-specific manner
Source: Sci Rep. 2018 May 15;8:7547. doi: 10.1038/s41598-018-25762-5 (PMC5953988; doi:10.1038/s41598-018-25762-5)
Supplement: Supplementary file 1 — Supplementary Information [file 41598_2018_25762_MOESM1_ESM.doc]

**Supplementary Information**

**Intrauterine smoke exposure affects lung function, pulmonary transcriptomes, and in particular insulin-like growth factor (IGF)-1 in a sex-specific manner**

**Stefan Dehmel^#^, Petra Nathan^#^, Sabine Bartel^2^, Natalia El-Merhie^2^, Hagen Scherb^3^, Katrin Milger^1^, Gerrit John-Schuster^1^, Ali Oender Yildirim^1^, Machteld Hylkema^4^, Martin Irmler^5^, Johannes Beckers^5,6,7^, Bianca Schaub^9^, Oliver Eickelberg^1^, Susanne Krauss-Etschmann^2,10*^**

^1^ Comprehensive Pneumology Center, Institute of Lung Biology and Disease, Helmholtz Zentrum Muenchen, Member of the German Research Center for Lung Research, Neuherberg, Germany.

^2^ Early Life Origins of Chronic Lung Disease, Research Center Borstel, Leibniz-Center for Medicine and Biosciences, Member of the German Research Center for Lung Research, Borstel, Germany.

^3^ Institute of Computational Biology, Helmholtz Zentrum Muenchen - German Research Centre for Environmental Health, Neuherberg, Germany.

^4^University of Groningen, University Medical Center Groningen, Department of Pathology and Medical Biology, GRIAC Research Institute, Groningen, The Netherlands.

^5^Institute of Experimental Genetics, Helmholtz Zentrum Muenchen, Neuherberg, Germany.

^6^Technische Universität München, Chair of Experimental Genetics, 85354 Freising, Germany.

^7^German Center for Diabetes Research (DZD), 85764 Neuherberg, Germany.

^8^Department of Pulmonary and Allergy, University Children's Hospital Munich, LMU Munich, Munich, Germany, Member of the German Research Center for Lung Research.

^9^Institute for Experimental Medicine, Christian-Albrechts-Universitaet zu Kiel, Niemannsweg 11, D- 24105 Kiel, Germany.

Stefan Dehmel and Petra Nathan contributed equally to this work (^#^).

Correspondence and requests for materials should be sent to S.K.E (^*^)

([skrauss-etschmann@fz-borstel.de](mailto:skrauss-etschmann@fz-borstel.de)).

**Materials and Methods**

**Sex determination of fetal mice.** For sex determination of mouse embryos, a PCR-based approach exploiting an intron difference of the X chromosomal *Kdm5c* gene and its Y chromosomal homolog Kdm5d (Agulnik *et al.*, Mamm Genome 8, 134-138, 1997, [PMID 9060413](http://www.pubget.com/paper/9060413)) was used. To this end, mouse tail clips were collected from E18.5 fetal mice and digested overnight with Proteinase K (Merck, Darmstadt, Germany). Genomic DNA from the supernatant of the digest was precipitated and 100 ng were used as template in PCR reactions with a final volume of 12 µl. Primer sequences for *Kdm5c/d* were 5’-GGCTGACTACTTCAACATGCCT-3’ and 5’-CCACTGCCAAATTCTTTGGA-3’. PCR reactions were carried out using a MasterCycler Gradient (Eppendorf, Hamburg, Germany) PCR machine with 35 cycles of amplification. PCR products were separated by agarose gel electrophoresis. A band size of 255 bp indicated presence of X chromosomal *Kdm5c*, whereas a band size of 226 bp indicated presence of Y chromosomal *Kdm5d*.

**RNA Isolation for qRT-PCR from murine lungs.** Total RNA including small RNAs was isolated using a bead-based tissue homogenization method (MagNaLyzer, Roche, Mannheim, Germany) using the miRNeasy Kit (Qiagen, Venlo, Netherlands) according to the manufacturer’s protocol. RNA quality and quantity were controlled via spectrometry (ND-100; NanoDrop Technologies, Wilmington, USA), capillary electrophoresis (Bioanalyzer Agilent 2100; Agilent Technologies, Santa Clara, USA) and standard gel electrophoresis. Only high quality RNA samples (RIN> 7; 260/280 ratio > 1.8; no degradation as detected by RNA agarose gel) were used for further analyses. For pediatric samples, total RNA was isolated with TRI Reagent (Invitrogen, Carlsbad, USA). One μg of RNA was reverse transcribed according to the manufacturer's instructions (Qiagen, Venlo, Netherlands).

**Single qRT-PCR of murine transcripts.** One µg RNA was transcribed using QuantiTect Reverse Transcription Kit (Qiagen, Venlo, Netherlands) according to manufacturer’s protocol. Specific expression patterns (**Supplementary Table S5**) were analyzed in 12 ng cDNA with the SybrGreen II Master Mix (Roche, Mannheim, Germany) on a LightCycler 480 II (Roche, Mannheim, Germany) using TATA binding protein (Tbp) as a reference gene.


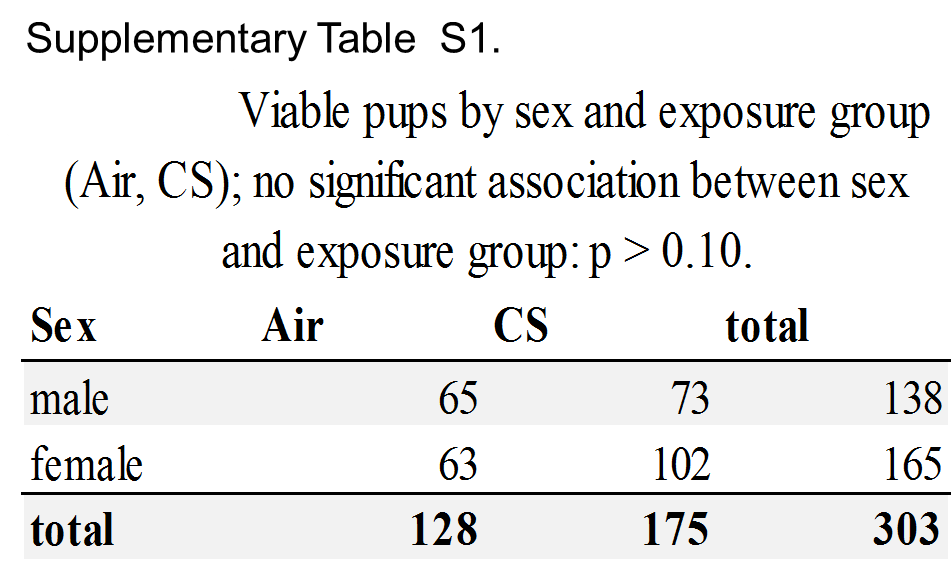


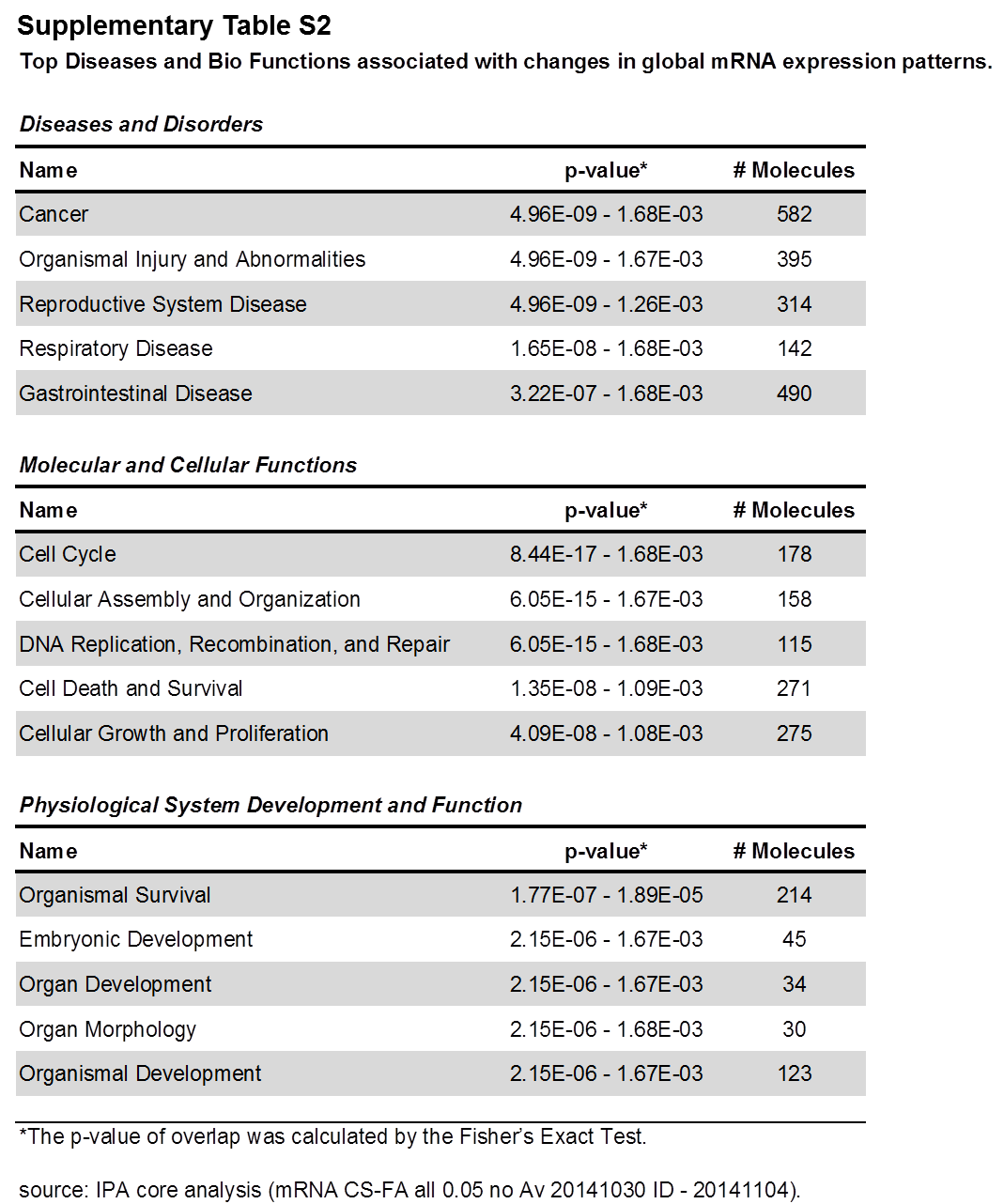


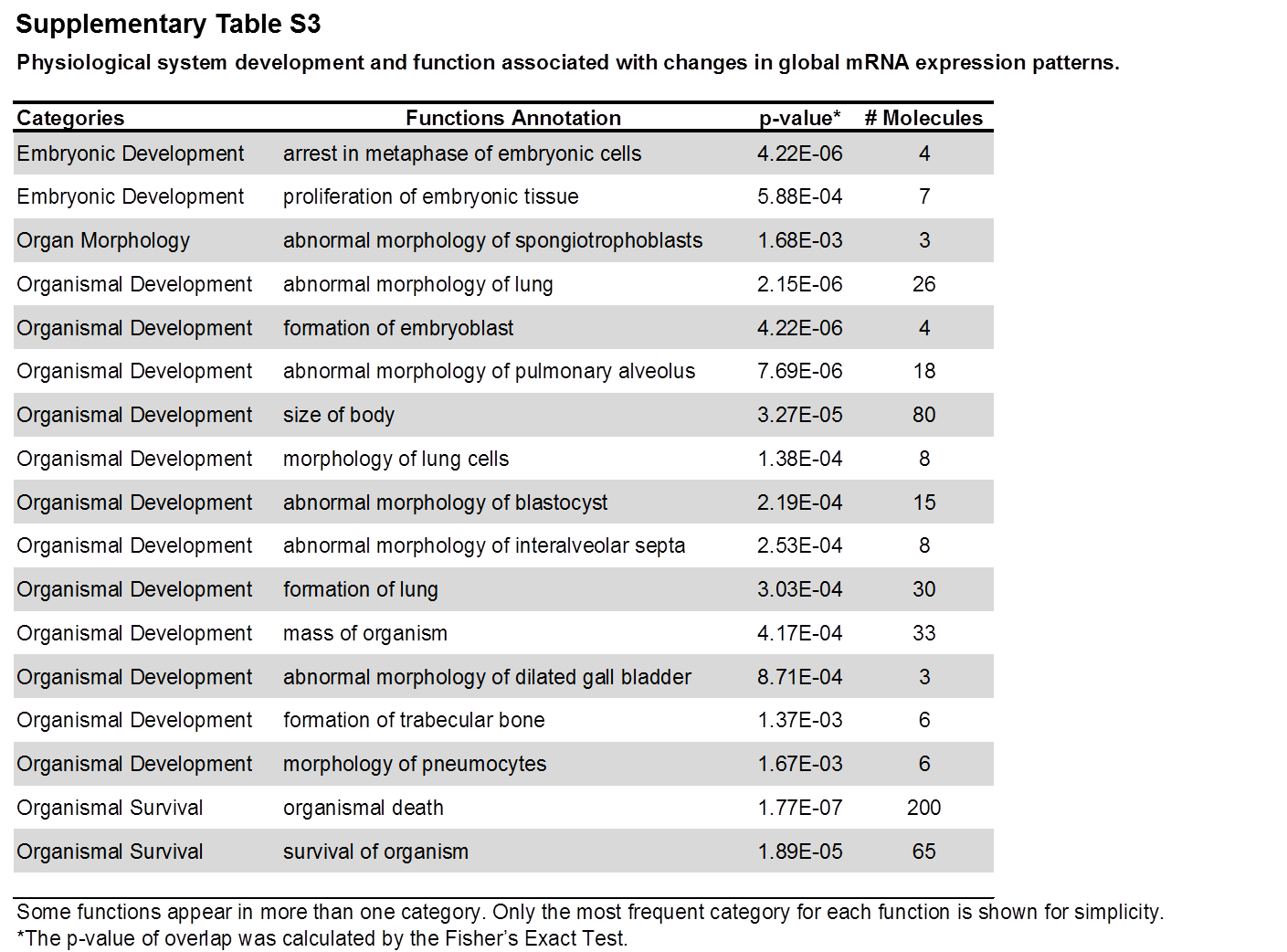


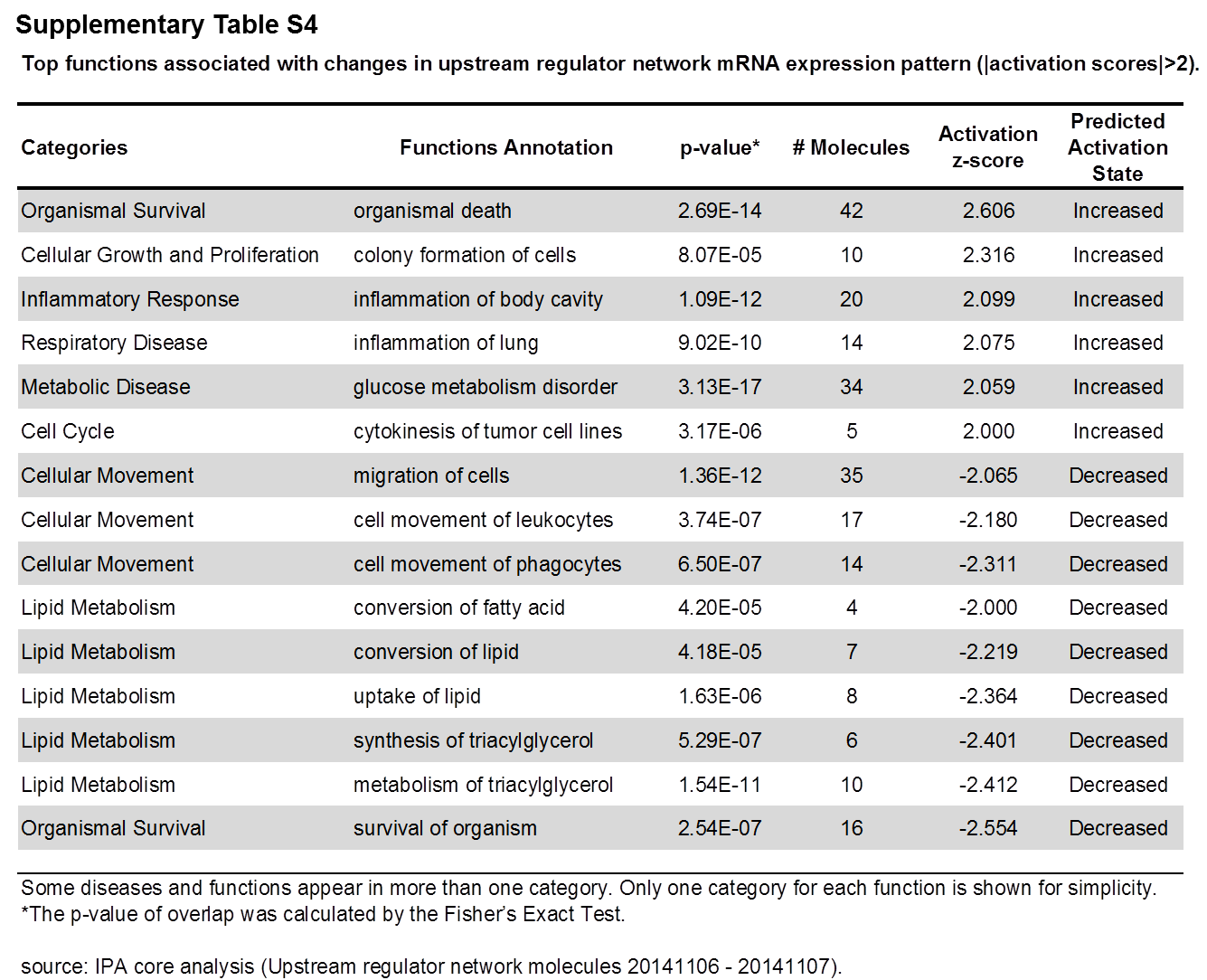


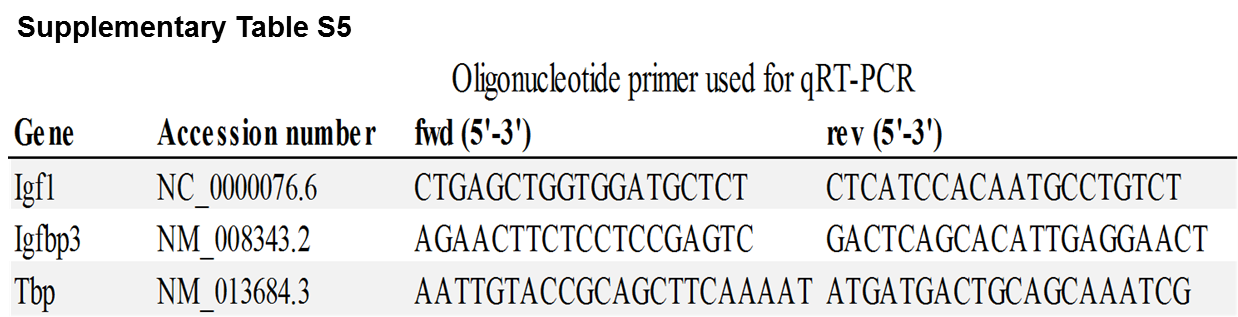


**
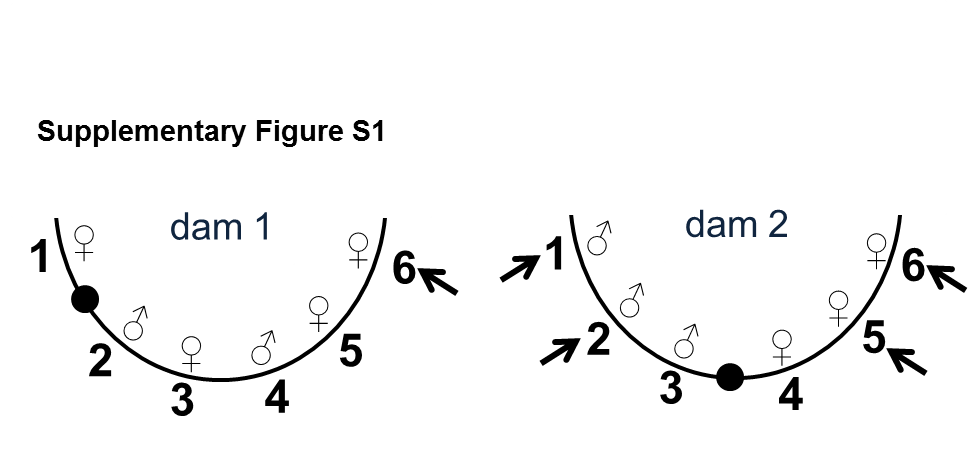
Supplemetary Fig. S1. Schematic representation of *in utero* position and selection of E18.5 fetuses for gene expression profiling.**

Semicircles indicate murine uteri while numbers represent individual fetuses and their sex (determined by genotyping). Dots (•) indicate position of visible resorptions. To minimize confounding effects due to hormonal exposure, only fetuses with neighbors of the same sex were selected (arrows) for profiling.

**
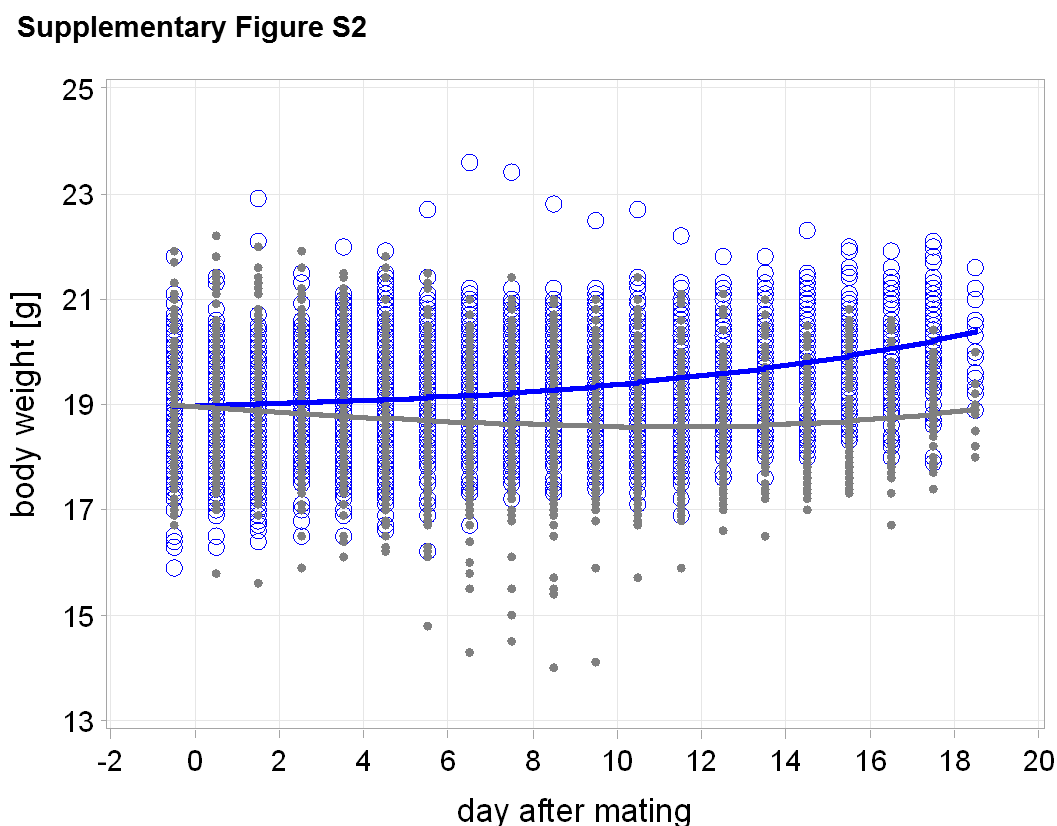
**

**Supplementary Fig. S2.** Weight trends of non-pregnant control mice (blue) and non-pregnant CS-exposed mice (grey); CS-exposed mice lag behind in weight gain by 80 mg/d (95%-CI: 75-84), Graphs depicts all observed values, especially the min and max values for each time point (open circles), and the corresponding optimum polynomial model fits (lines); p<0.0001.

**
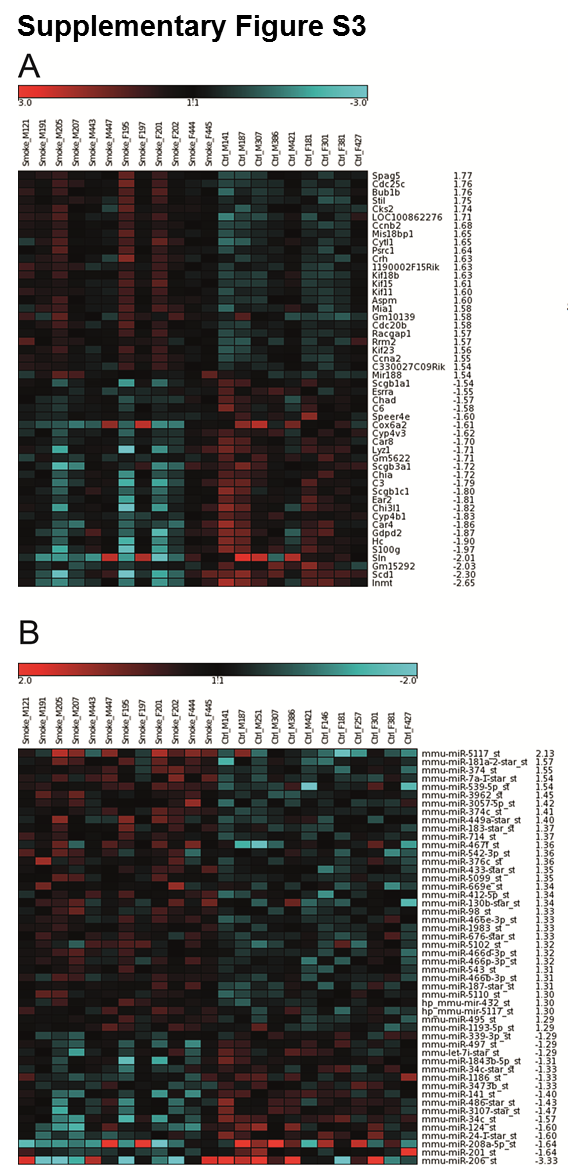
**

**Supplementary Fig. S3. Heatmaps and clustering of gene expression profiling**

Gene expression profiling with Affymetrix GeneChips® of lung homogenate from pubs after intrauterine AIR or CS-exposure. Expression profiles of the top 50 regulated genes for mRNA (**A**) and miRNA (**B**).
